# The effect of post-exercise caffeine and chlorogenic acid supplementation on blood glucose disposal and insulin sensitivity

**DOI:** 10.1186/1550-2783-10-S1-P2

**Published:** 2013-12-06

**Authors:** Jason R Beam, Ann L Gibson, Chad M Kerksick, Carole A Conn, Ailish C White, Christine M Mermier

**Affiliations:** 1 Department of Health, Exercise, and Sports Sciences, University of New Mexico, Albuquerque, NM 87131, USA; 2 Department of Individual, Family, and Community Education, University of New Mexico, Albuquerque, NM 87131, USA

## Background

Caffeine and chlorogenic acid are two compounds in green coffee beans that alter blood glucose disposal and insulin sensitivity. Caffeine has been shown to decrease glucose disposal and insulin sensitivity when taken 60 minutes prior to an oral glucose tolerance test in humans, whereas chlorogenic acid has been shown to increase glucose disposal and insulin sensitivity in humans. The purpose of this study was to investigate the effect of ingesting caffeine with dextrose or chlorogenic acid with dextrose immediately after an exhaustive bout of cycling on blood glucose and insulin disposal when compared to ingesting dextrose alone.

## Methods

Ten moderately to highly trained male cyclists (26±5 years; 179.9±5.4 cm; 77.6±13.3 kg; BMI: 24.0±4.3 kg·m^-2^; VO_2_ peak: 55.9±8.4 ml·kg^-1^·min^-1^) participated in this study. Each participant completed three experimental trials in random order the morning after abstaining from food, caffeine, and chlorogenic acid supplements for 12 hours. Each trial consisted of a 30-minute high intensity bout of cycling at 60% of peak power output (~90% HR max). Immediately after the exercise, each participant consumed 5 mg·kg^-1^ body weight of caffeine plus 75 g of dextrose (CAF), 5 mg·kg^-1^ body weight of chlorogenic acid plus 75 g of dextrose (CGA), or 5 mg·kg^-1^ body weight of dextrose plus 75 g dextrose (PLA). Blood was drawn to measure glucose and insulin immediately before exercise, immediately after exercise, every 15 minutes during the first hour of passive recovery, and every 30 minutes during the second hour of recovery. The blood glucose and insulin area under the curve (AUC) and Matsuda insulin sensitivity index (ISI) were calculated for each trial. Data were analyzed using ANOVAs with repeated measures and Pearson correlations (α=.05).

## Results

There were no significant time-by-treatment effects for blood glucose and insulin. The two-hour glucose and insulin AUCs, respectively, for the CAF (658±74 mmol/L and 30,005±13,304 pmol/L), CGA (637±100 mmol/L and 31,965±23,586 pmol/L), and PLA (661±77 mmol/L and 27,020±12,339 pmol/L) trials were similar (p > .05). The ISI for the CAF (9.7±5.2), CGA (12.1±7.9), and PLA (10.0±7.3) trials were also not significantly different (p > .05). There was substantial inter-subject variability in glucose and insulin responses during the three trials; this likely contributed to the non-significant findings. Body mass index was highly related to insulin AUC for the CAF (r=.71), CGA (r=.80), and PLA (r=.73) trials. Relative VO_2_ peak was inversely and moderately-to-highly related to insulin AUC for the CAF (r=-.82), CGA (r =-.63), and PLA (r=-.63) trials.

## Conclusion

Caffeine and chlorogenic acid may affect the body’s ability to regulate post-exercise insulin-mediated glucose transport into the exercised skeletal muscle through different mechanisms; however more research is warranted to verify this hypothesis. The heterogeneity of our sample highlights the inter-individual variability in post-exertional response to caffeine and chlorogenic acid when dosage is based on body weight. Consequently, we recommend that future investigations of glucose tolerance and insulin sensitivity utilize a sample that is homogenous in body composition and training status.

